# Incidental Parathyroid Disease during Thyroid Surgery: Should We Remove Them?

**DOI:** 10.5402/2011/962186

**Published:** 2011-04-20

**Authors:** S. Helme, A. Lulsegged, P. Sinha

**Affiliations:** Department of Surgery, Princess Royal University Hospital, Farnborough Common, Orpington, Kent BR6 8ND, UK

## Abstract

*Aim*. Despite an incidence of parathyroid “incidentalomas” of 0.2%–4.5%, only approximately 135 cases have been reported in the literature. We present eight patients in whom an incidental abnormal parathyroid gland was found during routine thyroid surgery. We have reviewed the literature and postulate whether these glands could represent further evidence of a preclinical stage of primary hyperparathyroidism. *Methods*. A retrospective analysis of all 236 thyroid operations performed by a single surgeon was performed to identify patients in whom abnormal parathyroid tissue was removed at surgery. *Results*. 8/236 patients (3.39%) had a single macroscopically abnormal parathyroid gland removed and sent for analysis. Seven patients were found to have histological evidence of a parathyroid adenoma or hyperplasia. None of the patients had abnormal serum calcium detected preoperatively. Postoperatively, four patients had normal calcium, three had temporary hypocalcaemia and one refused followup. No patients had recurrent laryngeal nerve impairment. *Conclusions*. Despite the risk of removing a histologically normal gland, we believe that when parathyroid “incidentalomas” are found during surgery they should be excised and sent for histological analysis. We have found this to be a safe procedure with minimal morbidity to the patient. As the natural history of primary hyperparathyroidism is better understood, these glands found in normocalcaemic patients may in fact represent the early or preclinical phase of the disease. By removing them at the original operation, the patient is saved redo neck surgery with its high complication rate as or when clinically apparent primary hyperparthryoidism develops in the future.

## 1. Introduction

Increasingly, patients with primary hyperparathyroidism are being identified at an earlier, asymptomatic stage of the disease. The reasons for this are multifactorial and include the development of more sensitive parathyroid hormone assays and increased screening of patients with measurement of PTH and serum calcium, for example, in a bone clinic. It is therefore, perhaps, not coincidental that endocrine surgeons are identifying macroscopically abnormal parathyroid tissue during thyroid operations in patients not known to have calcium disorders.

Investigation of thyroid disease often includes ultrasonography of the neck and diagnostic blood tests, and in some cases, coexistent thyroid and parathyroid disease may be noted. Discovery of both pathologies preoperatively involves clinical suspicion, radiological findings, or biochemical tests. Patients with the suspicion of PT disease can be fully investigated prior to their thyroid operation and if necessary have surgery to deal with both pathologies at the same time.

However, in our practice, we have noted a series of patients in whom parathyroid pathology was discovered incidentally during thyroid surgery. These patients were found to have a single macroscopically abnormal parathyroid gland on routine thyroid dissection without preoperative suspicion of concomitant parathyroid disease.

We describe the cases encountered and review the literature on the subject.

## 2. Methods

A retrospective analysis of all 236 thyroid operations performed by a single surgeon was performed to identify patients in whom unsuspected abnormal parathyroid tissue was removed at surgery. When such a gland was found, the remaining parathyroid glands were examined to ensure they were macroscopically normal, and the abnormal gland was excised and sent for histological examination.

## 3. Results

Between 2003 and 2008, our unit performed 236 thyroid operations-either total thyroidectomy or lobectomy. In eight patients (3.3%), a single macroscopically abnormal parathyroid gland was identified and excised during surgery. Seven (2.96%) of these were subsequently confirmed on histology to have either a parathyroid adenoma or hyperplasia. Seven patients were female, one was male, and the age range was 30–66 years. None of the patients had renal failure, a history of irradiation to the neck, or was taking regular Lithium. Three patients were subclinically thyrotoxic with depressed TSH but normal T4 levels.

Six of our patients had serum calcium levels checked preoperatively and these were all in the normal range, measuring from 2.19 to 2.50 mmol/l. Two patients did not have preoperative calcium checks, and none of our patients had preoperative parathyroid hormone (PTH) or vitamin D levels, as parathyroid disease was not suspected before surgery. Unfortunately, intraoperative PTH measurement is not available in our institution, so these measurements could not be taken.

Indications for thyroid surgery were pressure symptoms with multinodular goitre in four patients (A, B, F, and H), toxic multinodular goitre in two (D and E), Graves' disease in one (C), and a colloid nodule in the other (G)—see Table 1. 

The weight of the parathyroid glands removed ranged from 163 mcg to 844 mcg and the size ranged from 8 × 7 × 3 mm to 20 × 15 × 5 mm. 

Histopathological examination of the parathyroid tissue showed adenoma in three patients (A, E, and F), hyperplasia in one (C), features indistinguishable between adenoma and hyperplasia in three patients (B, D, and H), and normal parathyroid tissue in one patient (G). However, parathyroid adenomas are usually diagnosed on macroscopic appearances (single or double gland) rather than on histological findings. None of our patients had more than one abnormal gland detected on four-gland exploration.

Postoperatively, four patients had normal serum calcium levels, and three required oral supplementary replacement therapy for a few weeks postoperatively. One patient was discharged on Calcichew and 1-alpha calciferol but has refused postoperative followup or blood tests. 

## 4. Discussion

Primary hyperparathyroidism is a relatively common disease with incidences of between 1 : 200 and 1 : 1000 [1]. It is more common in women and in patients with a history of radiation to the neck [2]. 

Parathyroid disease is often diagnosed when a raised calcium level is found on “routine” blood tests for other medical problems. Usually, these patients are asymptomatic or suffer from nonspecific symptoms of hypercalcaemia such as fatigue, weakness, loss of appetite, nausea, and constipation. Less commonly nowadays, patients may present with significant renal and skeletal complications. 

However, autopsy studies have found that 1.9–7.6% of people have enlarged parathyroid glands without biochemical or clinical abnormalities and a similar range has been reported by endocrine surgeons [1–12].

A common time to discover these “incidentalomas” is at the time of thyroid surgery when most surgeons attempt to identify the parathyroid glands to ensure they remain well perfused and are not excised with the thyroid specimen. Published papers quote an incidence of 0.2% to 4.5% of unsuspected parathyroid disease found at the time of thyroid surgery in a normal population [1, 6]. Surprisingly, however, only approximately 135 cases have been reported since the first case report in 1937—see Table 1.

If a surgeon encounters a macroscopically abnormal PT gland during thyroid surgery, he faces a dilemma. Should he remove the gland, presuming it to be pathological, or should he leave it in situ so that full investigations can be performed to confirm that the patient has parathyroid disease? There are risks in either scenario, and these must be balanced by the surgeon when deciding what to do.

The main risk of removing the gland is that the patient may suffer from postoperative hypocalcaemia requiring long-term calcium replacement. If this were to be a hyperfunctioning adenoma then this risk is probably justified as the patient's hyperparathyroidism is cured. However, if it proves to be a normal gland, then the patient may have postoperative morbidity without any clinical benefit.

However, if an abnormal gland is left in situ, then there is the risk of needing to perform “redo” surgery in the future to remove it. This situation may arise because the gland is overactive at the time of surgery and this was not picked up preoperatively or because the disease is in its preclinical or early phase and may develop into overt hyperparathyroidism with time. Redo surgery carries with it a much higher risk of complications, particularly of bleeding, of damage to the recurrent laryngeal nerve (<1% in primary surgery and up to 10% in redo surgery [13]), and of postoperative hypocalcaemia due to inadvertent injury to normal parathyroid tissue either at the original surgery or redo operation.

As shown in Table 2, 10% of patients described in the published “incidentaloma” papers suffered from temporary postoperative hypocalcaemia and 1.5% required permanent supplementation. These figures are no higher than those seen in patients undergoing “routine” thyroid surgery, where incidence of temporary hypoparathyroidism after total thyroidectomy is quoted at 2–53% [14]. As there are four parathyroid glands, careful surgery to identify and preserve the remaining glands should not result in significantly increased risk of hypocalcaemia. Accidental removal of normal parathyroid tissue during thyroid surgery occurs in 6–21% cases of thyroid surgery, and for the most part studies, have not reported an increase in postoperative hypocalcaemia in such patients [15–19]. Treatment for those who do become hypocalcaemic, either temporarily or permanently, is relatively simple with oral calcium and vitamin D if necessary.

## 5. Natural History of the Disease

More is being discovered about the natural history of primary hyperparathyroidism, and this information may help in deciding the best course of action when discovering “incidentalomas” during thyroid surgery.

In 1993, Hellman et al. examined the structure and function of eleven enlarged parathyroid glands found in nine patients undergoing thyroid surgery with normal calcium levels. Although he found that only 8 out of 11 were microscopically abnormal, further examination with monoclonal antibodies and measurement of cytoplasmic calcium concentration showed that all but one were also functionally abnormal [7].

Since then, two groups, Carnaille et al. and Abboud et al., have examined the histological and biochemical features of enlarged parathyroid glands found incidentally in normocalcaemic patients and compared them to glands removed for overt primary hyperparathyroidism. “Incidentalomas” were found to occur in younger patients, to be lighter in weight, and to be biochemically and pathologically less hyperfunctioning, suggesting that these glands may represent an early stage of parathyroid disease [8, 12]. 

Development of accurate biochemical tests for parathyroid hormone, as well as increased awareness of skeletal health with the use of bone profiling and density scans, has led to an increased diagnosis of patients with primary hyperparathyroidism. In the past, patients were diagnosed when symptoms developed, but today, 80% of patients with overt disease are asymptomatic [20]. 

Endocrinologists are noticing an increase in the number of patients who have raised parathyroid hormones but normal calcium levels, and this leads to the question of whether a preclinical phase of disease exists [20–22]. Such patients are labelled as having “normocalcaemic hyperparathyroidism” in which calcium levels are normal but PTH is elevated. For accurate diagnosis, it is important to exclude other causes such as hypercalciuria, vitamin D deficiency, renal impairment, and bone disease, for example, Paget's disease in such a biochemical scenario. 

Unfortunately, in our case series, the patients had unexpected findings of parathyroid pathology. This meant that a thorough workup for parathyroid disease, such as preoperative measurement of PTH and vitamin D levels, was not performed. However, the nature of an incidentaloma is that it is not suspected prior to its discovery, so all of this information may not be available to the surgeon. Intraoperative PTH measurement would have allowed us to ascertain whether these patients had raised PTH levels associated with the enlarged gland, but this was not a facility available to us. Patients who suffer from Graves' disease can have macroscopically enlarged parathyroid glands secondary to their thyroid dysfunction. Three out of patients had subclinical thyrotoxicosis (one had Graves' disease and two had toxic nodules), but all three had free-T4 levels in the lower half of the normal range. This could have been a causative factor for the findings in some of our patients.

Given that coincidentally discovered abnormal parathyroid glands could represent early primary hyperparathyroidism, it is important to understand the progression of surgically untreated mild hyperparathyroidism before recommending routine removal of these glands.

The paper by Rubin et al. currently reports the longest followup of patients with untreated primary hyperparathyroidism. 57 of 116 patients were originally randomised to no surgery, and 49 of these patients were asymptomatic. At 10 years, the group reported the patients' results and showed evidence of disease progression in 25%. The followup study, published in 2008, reported that at 15 years, this had increased to 37% of asymptomatic patients developing criteria for surgery [23, 24].

In another paper, 53 patients with mild asymptomatic primary hyperparathyroidism were randomised to surgery or routine followup. Those randomised to surgery had a significant benefit on bone mineral density and quality of life and psychological well-being scores [25]. 

Silverberg and Bilezikian studied patients who had raised parathyroid hormone but normal calcium levels. Their paper centred on the observation that many patients have stable disease once primary hyperparathyroidism has been diagnosed, raising the question as to when in the disease course the biochemical and bony changes develop. They propose that “normocalcaemic” hyperparathyroidism is in fact the first phase of a biphasic disease, and it is in this period that the important biochemical and metabolic changes occur [22].

## 6. Screening

Denizot et al. looked at screening for parathyroid disease in patients undergoing thyroid surgery. Their group looked prospectively at 748 patients due to have thyroid surgery and screened them with preoperative serum calcium levels. If these were high or at the higher end of normal, then a parathyroid hormone level was measured. Nine patients (1.2%) were positively screened and all had parathyroid adenomas found at surgery. Of these, one-third needed specific dissection to find the abnormal gland, and it was deemed that these glands would have been missed at “routine” surgery. However, of the 739 patients who had negative screens, 12 were found to have “incidentalomas” and two patients had a missed diagnosis of primary hyperparathyroid disease discovered postoperatively [9]. 

Given what has been described above regarding a preclinical or first phase of hyperparathyroidism, maybe the screening tool above could have been improved by measuring parathyroid hormone rather than serum calcium. This is much more costly, so, for this to be a viable option, firm conclusions should probably be made regarding the medical and cost benefit of identifying these patients before they proceed for thyroid surgery. If the model outlined by Silverberg in 2003 is to be believed, then identifying these patients in the first phase and offering them a surgical cure will be to catch the disease in a destructive phase. Obviously, it would remain to be seen if identifying the offending gland in its preclinical phase is possible with the current localisation methods available.

## 7. Conclusions

Most endocrine surgeons will come across abnormal parathyroid glands when performing thyroid surgery. Despite the risk of removing a histologically normal gland, we believe that when parathyroid “incidentalomas” are found during surgery, they should be excised and sent for histological analysis. We have found this to be a safe procedure with minimal morbidity for the patient.

As the natural history of primary hyperparathyroidism is better understood, the abnormal glands found in normocalcaemic patients could represent the early or preclinical phase of the disease. However, our patient numbers are small, and there are no “control” cases to assess whether or not such patients might go on to develop primary hyperparathyroidism.

By removing these glands at the original operation, patients may avoid the need for redo neck surgery with its high complication rate if clinically apparent primary hyperparathyroidism were to develop in the future. 

Perhaps we should be more astute in trying to detect this patient population prior to surgery by keeping a high clinical suspicion of coexisting disease or even screening patients for parathyroid dysfunction prior to their thyroid surgery.

## Figures and Tables

**Table 1:** Histological analysis of the parathyroid tissue excised.

Patient	Age	Sex	Thyroid operation	Preoperative TSH/fT4	Parathyroid histology	Weight	Size (mm)	Preoperative corr. calcium	Postoperative corr. calcium	Hypo-calcaemia
A	40	F	Total	1.06/14.0	Adenoma	0.319 g	15 × 10 × 15	Not done	1.96	Temporary
B	40	F	Total	0.99/14.7	Adenoma/hyperplasia	0.501 g	21 × 7 × 6	2.19	2.02	Pt refused followup
C	30	F	Total	2.63/8.8	Hyperplasia	0.163 g	8 × 7 × 3	2.26	1.89	Temporary
D	66	M	Total	46.3/5.7	No specific type	0.6 g	30 × 8 × 5	Not done	2.31	No
E	50	F	Total	0.01/12.1	Adenoma	0.319 g	10	2.33	2.28	No
F	60	F	Total	0.22/12.8	Adenoma	<1 g	17 × 7 × 5	2.32	1.80	Temporary
G	40	F	Left lobectomy	0.9/13.9	Normal histology	Not stated	10 × 5	2.29	2.25	No
H	57	F	Total	0.16/13.5	Adenoma/hyperplasia	0.844 g	20 × 15 × 5	2.50	2.14	No

Normal ranges: TSH: 0.35–5.50 mu/l, fT4: 9.4–22.7 pmol/l, corr. Ca 2.15–2.60 mmol/l.

**Table 2:** Reported abnormal parathyroid glands discovered incidentally at thyroid surgery.

	Incidentalomas/thyroid operations	% total thyroid cases		Postoperative hypocalcaemia
1993 Hellman et al. [7]	8/594	1.35%		Unknown
1992 Katz and Kong [1]	36/800	4.5%		2/36 permanent
1996 Carnaille et al. [8]	26/4697	0.6%		0/26
1981 Prinz et al. [2]	8/23	34.8%	Irradiated pts	6/8 temporary
2001 Marchesi et al. [5]	3/1400	0.2%		1/3 temporary
2002 Denizot et al. [9]	12/739	1.6%	3 had normal histology	Unknown
2005 Lokey et al. [6]	33/738	4.5%		6/33 temporary
2005 Schroff et al. [10]	5/421	1.19%		0/5
2005 Whineray Kelly et al. [11]	1	Case report		1 temporary
2008 Abboud et al. [12]	11/574	1.9%		Unknown
